# MicroRNAs and Uveal Melanoma: Understanding the Diverse Role of These Small Molecular Regulators

**DOI:** 10.3390/ijms21165648

**Published:** 2020-08-06

**Authors:** Karen Aughton, Helen Kalirai, Sarah E. Coupland

**Affiliations:** 1 Liverpool Ocular Oncology Research Group, Department of Molecular and Clinical Cancer Medicine, Institute of Systems, Molecular and Integrative Biology, University of Liverpool, Liverpool L7 8TX, UK; hkalirai@liverpool.ac.uk (H.K.); s.e.coupland@liverpool.ac.uk (S.E.C.); 2 Liverpool Clinical Laboratories, Liverpool University Hospital Foundation Trust, Liverpool L69 3GA, UK

**Keywords:** uveal melanoma (UM), miRNA, biomarkers

## Abstract

Uveal melanoma (UM) is a rare tumour of the eye, characterised by a high propensity to metastasise in half of all patients, most frequently to the liver. Although there are effective treatment options for the primary tumour, once metastasis has occurred prognosis is poor, with overall survival limited to months. Currently, there are no effective treatments for metastatic UM, despite the tumour having a well-defined signalling pathway to which many therapies have been directed. In an effort to develop novel treatment approaches, understanding the role of other signalling molecules, such as microRNAs, is fundamental. MicroRNAs (miRNAs) are small non-coding RNA molecules involved in posttranscriptional gene regulation, resulting in reduced target gene expression and subsequent protein translation. In UM, several dysregulated miRNAs have been proposed to play a functional role in disease progression, whereas others have been put forward as clinical biomarkers of high-risk disease following isolation from blood, plasma and exosomes. Most recently, analyses of large datasets have identified promising prognostic miRNA signatures and panels. This review navigates the plethora of aberrant miRNAs disclosed so far in UM, and maps these to signalling pathways, which could be targeted in future therapies for the disseminated disease.

## 1. Introduction

Uveal melanoma (UM), although the most common primary intraocular tumour, is a rare disease, affecting approximately 4–6 adults per million annually [1,2]. UM has a propensity for affecting those with light eye colour, fair skin and an inability to tan, among other factors; and the incidence in Europe has been shown to follow a north–south downward gradient with a greater frequency of affected individuals residing in northern Scandinavian areas [3,4]. Ocular melanomas account for 3–5% of all melanomas, and the majority (approx. 85%) of these are located in the uvea, with the remainder arising in the conjunctiva [5,6,7]. UMs are thought to arise from melanocytes in the uveal tract, comprising the iris, ciliary body and choroid; most UM occur in the choroid (90%) [8].

Although treatment of the primary UM by radiotherapy, surgical resection or enucleation is most often successful with little local recurrence, approximately 50% of patients will develop metastases for which there are no current effective therapies [9]. The metastatic niche is most frequently the liver (90%), although other organs including bone, lung and skin can be affected [10]. Once metastatic UM is detected, the prognosis for the patient is poor, leading to death occurring within months [10].

UM are characterised by a low mutational burden, with recurrent chromosomal losses and gains culminating in a relatively simple genetic landscape [11]. Historically, UM have been subdivided into those with low or high metastatic risk, based largely on somatic copy number alterations of chromosome 3 [12]. Tumours with a high metastatic risk have a loss of one copy of chromosome 3, termed monosomy 3 (M3), and those at low risk have the normal two copies, termed disomy 3 (D3) [13]. Other genetic aberrations, including gains of chromosome 8q [12,13,14], and inactivating mutations of BRCA1 associated protein (*BAP1*) gene have been correlated to a high metastatic risk and hence poor outcome [15,16,17,18,19,20,21]. Mutually exclusive mutations in two other genes, eukaryotic translation initiation factor 1A (*EIF1AX*) and splicing factor 3 subunit 1 (*SF3B1*), have allowed further subdivision of the low metastatic risk D3 group, with *EIF1AX* mutations associated with non-metastasising tumours [20,22], whereas *SF3B1* mutations are associated with a delayed metastatic onset (intermediate risk) [11,23].

UM are initiated by a gain-of-function driver mutation in *GNAQ* or *GNA11* G-protein coupled receptors in almost 80% of cases [24,25,26], most frequently at the Q209 position and less frequently at the R183 position. This results in constitutive activation of the Gα downstream signalling cascade including activation of MAPK, PI3K-Akt-mTOR and Hippo (including YAP-TAZ) pathways [27,28,29,30,31,32]. A number of signalling cascade molecules, such as mitogen-activated protein kinase kinase (MEK) (selumetinib [33,34,35,36], trametinib [37]) and protein kinase C (PKC) (AEB071 [38,39]), have been targeted by therapeutics, with little or no change in patient overall survival [40,41,42]. Additionally, immunotherapy treatments targeting anti-CTLA-4 (ipilimumab [43]) and anti-PD-1 [44,45,46] have had low response rates; however, combination trials with immunotherapy agents, HDAC inhibitors (entinostat) and radioembolization are currently ongoing [40,47,48,49].

It is, therefore, imperative to explore other avenues of potential dysregulation within the UM landscape. Aberrant microRNA (miRNA) expression, frequently observed in a myriad of cancers, is also reported in UM. The studies undertaken in UM include those investigating (a) the functional roles of specific miRNAs in cell lines and tumour samples [50,51] and (b) the prognostic and diagnostic use of miRNAs, individually and in networks, from not only tissue samples but also from plasma and circulating exosomes [52,53], as well as larger data-mining in silico studies often utilizing and extrapolating the power of The Cancer Genome Analysis (TCGA) study [54,55,56]. However, at present, there is no clear consensus as to which of the identified miRNAs should be targeted for use either as diagnostic biomarkers or as potential druggable targets. This review explores aberrant miRNA expression in UM and maps these to signalling pathways that appear to regulate UM growth and could be exploited as future drug targets.

## 2. miRNA Role in Cancer

miRNAs are small, non-coding sections of RNA, approximately 21–25 nucleotides in length, which regulate gene expression in a sequence-dependent manner [57,58]. The first miRNA, lin-4, was discovered almost 30 years ago in the nematode *Caenorhabditis elegans* by Ambros et al., who reported sequence complementarity between lin-4 and the 3′ untranslated region (UTR) of the lin-14 gene [59,60]. This was followed in 2000 by the discovery of a second miRNA, let-7, regarded as a heterochronic gene required for developmental timing [61], which importantly was shown to retain evolutionary conservation between species [62]. There are now estimated to be approximately 2600 fully annotated miRNAs identified in the human genome [63], which are predicted to regulate anywhere between 30% and 60% of human mRNAs [64,65,66]. These small miRNA molecules are involved in the posttranscriptional gene regulation of key cellular processes, including apoptosis, differentiation, proliferation and cell cycle. Each miRNA carries a conserved seed sequence that is complementary to a miRNA response element (MRE) on the 3′ UTR region of target mRNAs [67]. Binding of the miRNA to MRE results most commonly in reduced gene expression by inducing translational repression, and mRNA deadenylation and decapping [68,69]; gene upregulation has also been reported and may result from direct action of miRNA binding or indirectly by relieving miRNA gene repression [70,71]. It is estimated that a single miRNA can target multiple genes, and a single gene can contain many MREs and be targeted by multiple miRNAs [72]. Synergism of miRNA regulation has been implicated in disease, and in oncogenesis [73], and allows modulation of complex regulatory networks of gene expression.

Dysregulation of miRNAs is frequently documented in most tumour types, with miRNAs most commonly downregulated [74]. This can be attributed to genetic loss, epigenetic silencing and defects in signalling pathways, resulting in reduced mature miRNA levels [75]. Studying miRNA in cancer is complicated, due to the diversity of tumours and cell lines; however, the ability to isolate miRNAs from cells, serum and plasma [76] means that they remain attractive molecules as biomarkers of both prognosis and diagnosis, therapeutic targets and epigenetic agents. The extensive capability of high throughput screening technologies and bioinformatic prediction analyses provides great tools for exploring the function and role of miRNAs; however, experimental validation will ultimately prove a miRNA’s worth.

## 3. miRNA Expression and Metastatic Risk in UM

**Figure 1:**
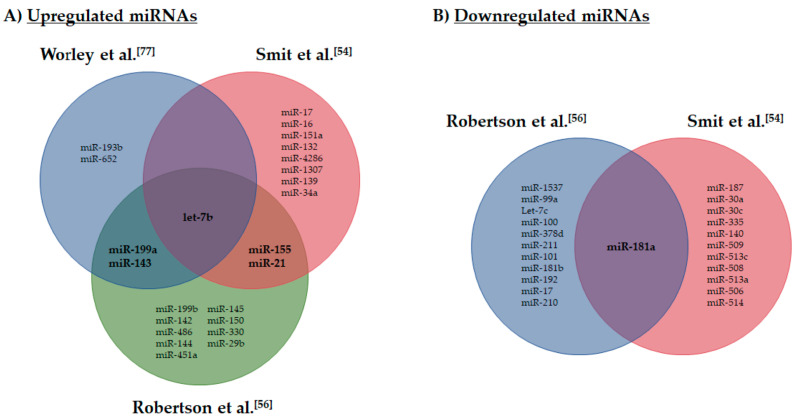
MicroRNAs (miRNAs) associated with an increased metastatic risk in uveal melanoma (UM). Evaluation of miRNA commonality was undertaken between three studies examining miRNA expression de novo in large well-defined UM sample sets. (**A**) Venn diagram of upregulated miRNAs associated with an increased metastatic risk. (**B**) Venn diagram of downregulated miRNAs associated with an increased metastatic risk. The miRNAs included in this analysis were obtained from Worley et al. (only upregulated miRNAs reported), Robertson et al. (miRNAs comparing cluster 3 + 4 with 1 + 2, fold change >2) and Smit et al. (all miRNAs found in high and low/intermediate metastatic risk comparisons). Overlapping miRNAs are in bold font and commonalities are identified for the upregulated miRNAs et-7b, 199a, 143, 155 and 21, and for the downregulated miRNA 181a. Only the mature miRNA identifiers were used within this comparative analysis.

The first insight into miRNA dysregulation in UM was provided by Worley et al., using microarray technology, with a view to identifying a prognostic biomarker of metastatic risk [77]. Having previously observed that gene expression profiling allowed UM to be separated into distinct groups correlating with prognosis [78], Worley and co-workers noted that miRNA expression also clustered UM into two distinct prognostic groups according to metastatic risk. They reported six upregulated miRNAs that could accurately distinguish low metastatic risk class 1 UM and high metastatic risk class 2 UM, and in particular they highlighted let-7b and miR-199a as major discriminators of high metastatic risk class 2 UM [78]. In a limited study by Radhakrishnan et al., the miRNA expression profiles of a single primary UM from a patient with metastatic disease were compared with that of a primary UM from a patient who had not developed metastatic disease [79]. Eleven miRNAs were identified that were present in the metastasising versus the non-metastasising primary UM [79]; none of these miRNAs were identified in more comprehensive expression studies analysing metastatic risk [54,56,77] (Figure 1).

Most recently, a TCGA-UM study, which analysed 80 primary UM samples, identified four main miRNA clusters that were clearly associated with chromosome 3 status, metastatic risk and corresponding DNA methylation profile [56]. It was demonstrated that miR-199a-3p/5p and let-7b-5p were highly expressed in M3-UM miRNA cluster 3 [56], supporting the data of Worley et al. [77]. In addition, M3-UM expressed lower levels of miRNAs located on chromosome 3 [56]. The wealth of information provided by the TCGA-UM study has allowed for larger data-mining in silico studies often using these data, and some of these are discussed later in this review.

Smit et al., combined in-house microarray miRNA expression data with data-mining of the TCGA mRNA dataset for predicted gene targets [54]. Data from 26 primary UM samples subdivided into metastatic risk subgroups on the basis of chromosome 3 status and mutational sequencing for *EIF1AX* and *SF3B1* (7 low risk, 12 intermediate risk, 7 high risk) were analysed for differentially expressed miRNAs; miR-17-5p, miR-21-5p and miR-151a-3p were differentially expressed between high-risk and low-/intermediate-risk groups [54]. Following gene target association using the TCGA-UM dataset (106 genes predicted), bioinformatic pathway analysis software revealed that the miRNAs interact with genes involved in cancer-related pathways including cell cycle regulation pathways, EIF2 and EGF signalling [54].

A similar approach was taken by Wróblewska et al., but this time combining TCGA miRNA data with miRNA validation in patient samples [80]. They identified a panel of 15 miRNAs differentially expressed between four TCGA-UM study patients (stage IIA tumours and without metastatic disease) and four TCGA-UM patients (stage IV with metastatic disease). Further validation of six of the miRNAs in 28 primary UM with no evidence of metastasis and 18 primary UM, where metastatic disease had developed at the time of follow-up, demonstrated that miR-592, miR-346 and miR-1247 were significantly increased, and that miR-506 and miR-513c were significantly decreased in the metastatic subgroup [80].

In contrast to the above studies, Larsen et al., found no association of miRNA expression with metastasis and survival in 26 primary UM patients, 15 of whom had died of metastatic disease [81].

To examine the commonality of the miRNAs reported to be associated with increased metastatic risk, we compared three studies that examined miRNA expression de novo in large clinically well-defined UM sample sets [54,56,77]. Interestingly, little overlap was noted for the miRNAs identified by each study (Figure 1), with similarities only being observed for let-7b, miR-199a, miR-143, miR-155 and miR-21 (upregulated miRNAs), and only for miR-181a (downregulated miRNAs). Differences in study design, tissue sample classifications and miRNA detection systems (microarray vs. RNA-Seq) may all contribute to the differences in miRNAs reported as significantly associated with an increased metastatic risk in UM. However, this lack of commonality between studies emphasises the need for functional validation of any proposed miRNAs as biomarkers in UM moving forward.

## 4. Functional Role of miRNAs in UM

When considering the functional role of miRNAs in UM, it is generally accepted that miRNAs will have either a tumour-suppressing or tumour-promoting role, and miRNA dysregulation is reflected by the expression levels of the miRNA in the tumour. An increasing number of studies are beginning to define the role of many different miRNAs in UM samples and cell lines, predominantly using in vitro systems, although some data stem from in vivo models. miRNA targets have been derived from in-house microarray expression data, or identified from studies in other cancer types, in particular skin melanoma, which may hold relevance to UM signalling pathways.

Generally, the format of these previous studies addresses the expression of the miRNA in UM cell lines and tissue, compared with melanocytes and “normal” (often adjacent) tissue, respectively. This is subsequently followed by cell proliferation, invasion, migration and cell cycle analyses with or without transfection of miRNA mimics and inhibitors. Bioinformatic predictions using miRNA databases are employed to determine downstream targets, which are confirmed using luciferase assays. Additional analyses include target gene expression levels in situ, and in response to miRNA mimics or inhibitors, again correlating this with effects on cell growth, invasion and migration to confirm the miRNA–gene relationship. While these studies have provided a large amount of information, certain limitations exist. For example, the UM cell lines used within the majority of studies are restricted to two or three of those that are less well characterised in the literature [82,83]. There are a large number of genetically characterised UM cell lines available, which better represent the disease and should be considered for use as a panel, to gain a more complete understanding of the target miRNA function. In particular, the use of UM cell lines with loss of nuclear BAP1 expression and/or chromosome 3 loss would be beneficial to understand their role in metastatic progression [84].

Nevertheless, the functional studies undertaken in UM examine both oncogenic and tumour suppressor miRNAs, providing key data for further evaluation. Oncogenic miRNAs downregulate tumour suppressor gene transcription, and are listed in Table 1. On the contrary, downregulation of tumour suppressor miRNAs results in UM progression by allowing their target oncogene to be subsequently upregulated. Tumour suppressor miRNAs are listed in Table 2.

### 4.1. miRNA as Oncogenes in UM

Oncogenic miRNAs in UM include the miR-181 family, comprising four miRNAs (miR-181a/b/c/d), which are transcribed from different gene loci. In UM, it was found that all miR-181 family members were highly homologous and able to regulate the effector gene *CTDSPL*, a phosphatase-like tumour suppressor gene that dephosphorylates Rb1 and regulates the RNA polymerase II transcription machinery [51]. All members of the miR-181 family were significantly overexpressed in UM and cell lines (including 92.1 cells); however, miR-181b was highlighted as the most effective regulator of the downstream gene *CTDSPL*. miR-181b repression of *CTDSPL* resulted in Rb protein phosphorylation and E2F1 transcription factor protein expression, with subsequent cell cycle progression [51].

Other oncogenic miRNAs include miR-155 [85], miR-20a [86], and miR-216a-5p that targets the hexokinase 2 (*HK_2_*) gene involved in cell metabolism [87]. Interestingly, two oncogenic miRNAs have been shown to target *PTEN* in UM, namely miR-454 [88] and miR-367 [89]. Both miRNAs were upregulated in UM cell lines and samples, and had significant effects on promoting cell proliferation, cell cycle, migration and invasion by directly targeting *PTEN*. Individually, miR-454 has been reported to be involved in hepatic stellate cell activation [90], while both miRNAs are involved in TGFβ signalling pathways via the *SMAD* family of genes [89,91]. These studies highlight that multiple miRNAs are able to target a single gene, and also that data from “single gene–single target” studies must be viewed in a wider context when trying to determine the overall miRNA landscape of UM.

### 4.2. miRNA as Tumour Suppressors in UM

miRNAs considered to be tumour suppressors in UM include members of the miR-34 family, to which multiple manuscripts are dedicated. The miR-34 family has been implicated in multiple solid tumour types (e.g., colorectal, breast, lung and liver carcinomas, as well as osteosarcomas [95]). In UM, miR-34a/b/c [50,96] are all downregulated and predicted to target c-Met directly, with other indirect targets, including p53, pAkt and numerous cell cycle proteins. Transfection of miR-34a/b/c resulted in target protein downregulation and reduction in cell proliferation, invasion and migration [50,96]. Follow-on studies of miR-34a have reported its involvement in the regulation of *LGR4*, a universal tumour promoter, and downstream epithelial-to-mesenchymal transition proteins such as E-cadherin, N-cadherin, vimentin, SNAIL and MMP2 [97], such that downregulation of miR-34a lends to activation of epithelial signalling [97].

Since the identification of the important role of the miR-34 family in UM, the first tumour-targeted miRNA drug, MRX34, has been used in a phase I clinical trial based on miR-34a [95]. Unfortunately, MRX34 was withdrawn due to immune-related adverse events; however, it highlights the advances being made in this field and the possibility of epigenetic modulating drugs being used in the future treatment of disseminated UM. Finally, miR-34a response elements (MREs) have been used to help direct a TRAIL-expressing adenoviral vector in UM cell lines and UM xenografts with high anti-tumour activity, thereby helping to improve specificity of drugs [98].

Most recently, Amaro et al., investigated the regulation of metastatic genes *ADAM10* and *c-Met* by miR-122 and miR-144 [99]. In this study, they confirmed downregulation of both miRNAs by microarray analysis in primary UM samples and cell lines, and analysed the TCGA database to validate their findings. Further analysis showed that overexpression of miR-122 and miR-144 reduced ADAM10 and c-Met protein expression resulting in reduced proliferation, migration and cell cycle progression [99].

### 4.3. Alternative Mechanisms of miRNA Regulation in UM

Regulation of miRNA by other epigenetic molecules, such as long-noncoding RNAs (lncRNAs), have also been shown to play a role in UM progression. Oncogenic miR-124 is regulated by lncHOXA11-AS, and targets EZH2-dependent repression of the cyclin dependent kinase inhibitor *p21* [93]. In addition, the tumour suppressor miRNA-224-5p is regulated by lncFTH1P3, targeting *Rac1 and Fizzled 5* [108], whereas miR-17-3p is regulated by lncPVT1, targeting *MDM2* [111] (these are denoted in Table 1 and Table 2 by an asterisk (*)). These provide evidence that epigenetic regulation of miRNAs by other mechanisms, adds an additional layer in the complexity of signalling pathway regulation.

## 5. miRNAs as Clinical Biomarkers of UM

When undertaking any miRNA scientific study, the ultimate goal will either be a functional outcome for potential epigenetic modification or drug targeting, or as a biomarker for prognosis and diagnosis. In UM, chromosomal mutations and genetic aberrations in the primary tumour can predict with relatively high accuracy whether a UM has a low or high risk of metastasis. When incorporated with other prognostic parameters, i.e., clinical and histological characteristics, expected survival for an individual patient can be predicted [113]. This is a robust approach that has been translated into the clinic; however, it requires tissue samples obtained by fine-needle biopsies, tumour resections or enucleations [114]. Therefore, there is a huge appeal in being able to detect dysregulated miRNAs non-invasively from plasma and serum samples of UM patients [115].

### 5.1. Blood Biomarkers in UM

Triozzi et al., looked into monitoring anti-angiogenic treatments in UM by detecting miRNAs in blood and associating them with circulating endothelial cells (CECs) and angiogenic proteins [116]. Blood samples from patients on an adjuvant therapy trial receiving dacarbazine followed by interferon-alfa-2b were used to detect the presence of a panel of nine plasma miRNAs. Of these miRNAs, miR-199a and miR-106a were shown to correlate with CEC pre-therapy. Furthermore, the plasma levels of these miRNAs and those of miR-216 and miR-16 were shown to change following treatment with interferon-alfa-2b, but not dacarbazine. Follow-on studies from this trial subsequently correlated circulating plasma miRNA levels with metastasis [117]. In a study of six UM patients, miR-20a, miR-125b, miR-146a, miR-155, miR-181a and miR-223 plasma levels were all shown to be increased at time of diagnosis of primary UM, compared with healthy controls (no ocular disease) [117]. At the time of metastasis, miR-20a, miR-125b, miR-146a, miR-155 and miR-223 plasma levels had increased, and miR-181a decreased [117]. In serum samples from UM patients undergoing enucleation, eight differentially expressed miRNAs were identified (miR-146a, miR-523 upregulated; miR-19a, miR-30d, miR-127, miR-451, miR-518f and miR-1274B downregulated) compared with normal controls [118]. Furthermore, miR-146a was validated in formalin-fixed paraffin-embedded (FFPE) UM samples and confirmed to be upregulated [118]. Finally, a study comparing plasma and UM sample miRNA expression confirmed three miRNAs to be significantly elevated and which were previously detected as increased in tissue microarrays of M3 UM compared with D3 tumours—namely, miR-92b, miR-199-5p and miR-223 [52].

Most recently, Stark et al. conducted a cross-sectional, multicentre study to determine whether circulating miRNAs in serum could help distinguish benign uveal nevi from UM [119], one of the remaining clinical diagnostic difficulties. Using a panel of 17 miRNAs, the authors discovered that miR-16, miR-145, miR-146a, miR-204, miR-211 and miR-363-3p all showed significant differences between uveal nevi and primary/metastatic UM [119]. In addition, miR-211 was able to distinguish metastatic UM from primary UM. When analysing the six miRNA distinguishers as a group, and after assigning diagnostic scores to the data and using multiple testing statistics with defined cut-offs that allowed categorisation of expression values as positive or negative for UM, the authors found that, when ≥4 miRNAs reached or exceeded their cut-off, they could successfully distinguish uveal nevi from UM [119].

### 5.2. Exosomal Biomarkers in UM

Other sources of miRNAs that have been studied are those present in exosomes. Exosomes are small (typically 30 to 100 nm), membrane-bound vesicles released by eukaryotic cells that carry a range of bioactive molecules including miRNAs [120]. Cancer cells have been reported to aberrantly release exosomes in order to communicate with their environment, thereby promoting cellular processes such as proliferation, angiogenesis and the preparation of the pre-metastatic niche [120,121].

In UM, exosomes were successfully isolated from the liver perfusate of patients with metastatic disease undergoing isolated hepatic perfusion [53]. Melan-A positive exosomes were found to be present in greater numbers in patients with metastatic UM than those isolated from healthy peripheral blood plasma controls. Exosomes derived from metastatic UM in the liver shared distinct miRNA clusters, which were different from exosomes isolated from tumour cell cultures; however, these did not include cultures from UM cells [53]. Another study examined exosomes isolated from the vitreous humour (VH) with miRNA profiles of serum and VH from UM patients and healthy controls. The study demonstrated that VH exosome miRNAs and VH miRNAs from UM patients shared similar expression profiles, but were distinct from miRNAs isolated from serum of the same patients [122]. Dysregulated miRNAs in VH and vitreous exosomes included upregulated miR-21, miR-34a and miR-146a, of which miR-146a was also upregulated in UM patient serum and exosomes [122].

## 6. In Silico Predictive Studies of miRNA Biomarkers in UM

The publication of the TCGA-UM study, which applied high-throughput sequencing of 80 primary UM samples to establish a large publicly available dataset of genomic data [56], has enabled extensive in silico data-mining by various groups. The output of these studies identifies a variety of miRNAs, sometimes with commonality between studies, but most often identifying novel miRNAs that are cited as predictive biomarkers of high-risk UM. As mentioned previously, the lack of concordance between studies can be attributed to differences in study design and data handling, and highlight the importance in further validating these miRNAs as predictive biomarkers. However, it is encouraging that there are multiple avenues with which to secure successful predictive biomarkers for future clinical use.

Falzone et al., published a study looking for prognostic miRNA biomarkers by stratifying UM patients into two groups; first by tumour stage (high-grade vs. low-grade) and subsequently by survival status (dead vs. alive) [55]. From this, they identified seven dysregulated miRNAs in common between the groups, including five downregulated (miR-514a-3p, miR-508-3p, miR-509-3-5p, miR-513c-5p and miR-513a-5p) and two upregulated (miR-592 and miR-199a-5p) [55]. Further analysis revealed that the most highly dysregulated miRNAs in the high-grade versus low-grade UM groups, which correlated with overall survival, were miR-506-514 cluster, miR-592 and miR-199a-5p [55]. Gene ontology analysis showed that genes associated with miRNAs highlighted in this study include those involved in the MAPK1 and PI3K-Akt pathways, known components of UM signalling, as mentioned above.

Other prognostic signatures identified include a nine-miRNA signature derived from dividing the TCGA-UM dataset randomly into a training set and a test set (40 per group) and using a Cox proportional hazard regression model and receiver operating curves (ROCs) to confirm signature accuracy [123]. The nine-miRNA signature includes upregulated levels of miR-195, miR-224, miR-365a, miR-365b, miR-452, miR-4709, miR-7702 associated with increased metastatic risk, as well as miR-513c and miR-873 with high expression that were associated with reduced metastatic risk; when combined, this signature was able to accurately distinguish UM patients at high and low metastatic risk [123].

## 7. Conclusions

In summary, a large number of varied miRNA studies have been undertaken to help define the miRNA expression profiles in UM, the functional role of some of these miRNAs and their predicted gene targets for use as future prognostic biomarkers or drug targets for epigenetic and small-molecule agents.

**Figure 2:**
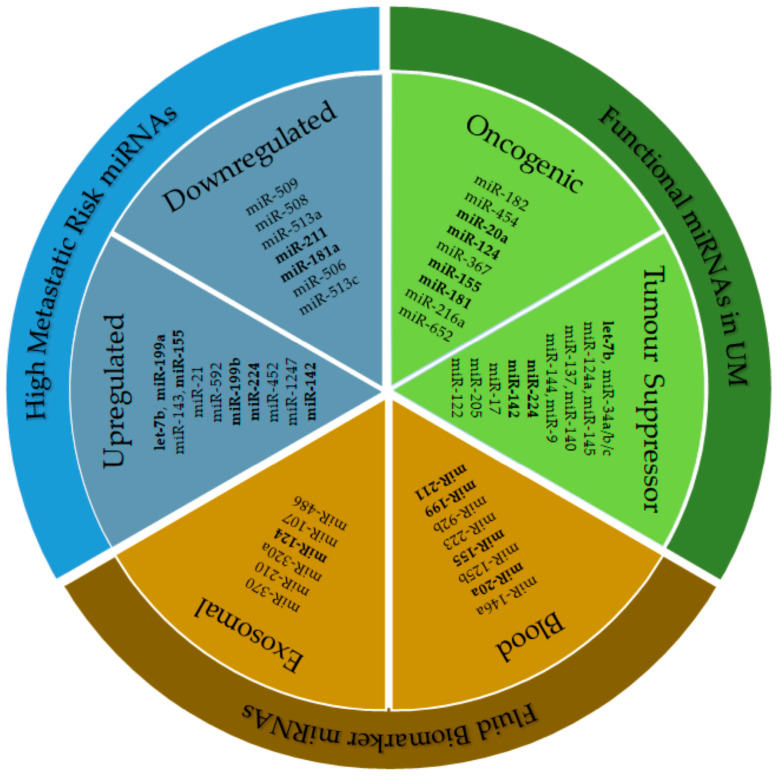
Selected miRNAs reported in uveal melanoma (UM) studies that have been examined functionally and are associated with disease progression or increased metastatic risk. These data include all functional miRNAs examined in Table 1 and Table 2, miRNAs identified as fluid biomarkers of disease progression or metastasis as well as miRNAs identified as associated with increased metastatic risk from both UM tissue samples and in silico data-mining studies. All data presented are significantly changed miRNAs in the study setting and have been cross-compared between studies to identify important miRNAs. Common miRNAs identified in the different study settings are in bold.

The range of miRNAs with assumed importance in UM progression and signalling is vast; however, some commonalities can be drawn from the data, and an overview is shown in Figure 2. Within this figure, miRNAs potentially associated with UM progression and metastatic risk and those already examined in functional studies are listed; miRNAs reported across multiple studies are highlighted in bold, and include those that are upregulated (miR-20a, miR-124, miR-155, miR-224, let-7b, miR-142 and miR-199) as well as those that are downregulated (miR-181a and miR-211). Bioinformatic pathway analyses combined with functional analyses indicate that cell cycle and translation pathway dysregulation are significant in UM; moreover, MAPK and PI3K-Akt signalling appear to be promising targets of multiple dysregulated miRNAs.

While these studies may highlight particular miRNAs that are involved in UM development and progression, it is important to emphasise that these studies need to be considered together, in order to fully understand the complex regulatory signalling of miRNAs in UM. On the one hand, functional analyses undertaken to date tend to be stand-alone observations, and do not address the miRNA networks known to be involved in cancer. On the other hand, bioinformatic analyses are often only predictive, and require functional validation in an in vitro setting. Experimental caveats also have to be considered, including the choice of UM cell lines and their genetic diversity. The genetic and chromosomal aberration data of the samples examined are sometimes not reported in the functional studies, and therefore their observations or conclusions may be oversimplified. Additionally, future studies may take advantage of emerging three-dimensional preclinical models in a range of genetically diverse UM cell lines, exploring the signalling role of miRNAs in a more clinically relevant setting [124].

This review has attempted to summarise the main findings of these numerous studies, emphasising where overlap occurs and suggesting a “roadmap” to direct future studies of miRNAs in UM.

## Figures and Tables

**Table 1:** Upregulated oncogenic miRNAs in UM.

miRNA	Target	Functions	Reference
miR-182	*MITF*, *BCL2*, *CyclinD2*	Proliferation, cell cycle, colony formation, migration, invasion. Doxorubicin sensitivity	[92]
miR-454	*PTEN*	Proliferation, colony formation, invasion, cell cycle	[88]
miR-20a	*-*	Proliferation, invasion, migration	[86]
miR-124 (lnc HOXA11-AS) *	*p21*	Proliferation, invasion, apoptosis	[93]
miR-367	*PTEN*	Proliferation, cell cycle, migration	[89]
miR-155	*NDFIP1*	Proliferation, invasion	[85]
miR-181	*CTDSPL*	Cell cycle	[51]
miR-216a-5p	*HK_2_*	Glycolysis, lactate production, ATP generation, ECAR, OCR	[87]
miR-652	*HOXA9*	Proliferation, migration	[94]

*: Denotes microRNAs (miRNAs) that are regulated by long-noncoding RNAs (lncRNAs). ATP – adenosine triphosphate, ECAR – extracellular acidification rate, OCR – oxygen consumption rate.

**Table 2:** Downregulated tumour suppressor miRNAs in UM.

miRNA	Target	Functions	Reference
miR-34a	*c-Met*	Proliferation, migration	[50]
miR-137	*MITF*, *CDK6*	Proliferation, cell cycle	[100]
miR-34b/c	*c-Met*	Proliferation, migration, cell cycle	[96]
miR-9	*NF-κB1*, *MMP2/9*, *VEGFA*	Proliferation, migration, invasion	[101]
miR-124a	*EZHZ*, *CDK4*, *CDK6*, *CCND2*	Proliferation, migration, invasion, colony formation, cell cycle Epigenetically regulated methylation and histone modification	[102]
miR-145	*IRS-1*	Proliferation, cell cycle, apoptosis	[103]
let-7b	*CyclinD1*	Radiosensitivity, cell cycle, proliferation	[104]
miR-144	*c-Met*	Proliferation, invasion	[105]
miR-137	*SRC1*, *2*, *3*	Proliferation, cell viability	[106]
miR-140 (lnc MALAT1) *	*Slug*, *ADAM10*	Proliferation, colony formation, migration, invasion	[107]
miR-224-5p (lnc FTH1P3) *	*Rac1*, *Fizzled 5*	Proliferation, migration, cell cycle	[108]
miR-224-5p	*PIK3R3-AKT3*	Proliferation, invasion, migration	[109]
miR-142-3p	*CDC25C*, *TGFβ1R1*, *GNAQ*, *WASL*, *RAC1*	Proliferation, invasion, migration, cell cycle	[110]
miR-34a	*LGR4*	Migration, invasion	[97]
miR-17-3p (lncPVT1) *	*MDM2*	Cell viability, invasion, migration, apoptosis, cell cycle, tumour volume	[111]
miR-145/205	*NRP1*	Proliferation, invasion	[112]
miR-122/144	*ADAM10/c-Met*	Proliferation, migration, cell cycle	[99]

*: Denotes miRNAs that are regulated by lncRNAs.
